# Nanocellulose Derivative/Silica Hybrid Core-Shell Chiral Stationary Phase: Preparation and Enantioseparation Performance

**DOI:** 10.3390/molecules21050561

**Published:** 2016-05-04

**Authors:** Xiaoli Zhang, Litao Wang, Shuqing Dong, Xia Zhang, Qi Wu, Liang Zhao, Yanping Shi

**Affiliations:** 1 Key Laboratory of Chemistry of Northwestern Plant Resources and Key Laboratory for Natural Medicine of Gansu Province, Lanzhou Institute of Chemical Physics, Chinese Academy of Science, Lanzhou 730000, China; lzuzxl@163.com (X.Z.); wlt@licp.cas.cn (L.W.); sqdong@licp.cas.cn (S.D.); xiazhang@licp.cas.cn (X.Z.); wuqi@licp.cas.cn (Q.W.); 2 University of Chinese Academy of Science, Beijing 100039, China

**Keywords:** nanocrystalline cellulose, chiral stationary phase, core-shell material, enantioseparation, high performance liquid chromatography

## Abstract

Core-shell silica microspheres with a nanocellulose derivative in the hybrid shell were successfully prepared as a chiral stationary phase by a layer-by-layer self-assembly method. The hybrid shell assembled on the silica core was formed using a surfactant as template by the copolymerization reaction of tetraethyl orthosilicate and the nanocellulose derivative bearing triethoxysilyl and 3,5-dimethylphenyl groups. The resulting nanocellulose hybrid core-shell chiral packing materials (CPMs) were characterized and packed into columns, and their enantioseparation performance was evaluated by high performance liquid chromatography. The results showed that CPMs exhibited uniform surface morphology and core-shell structures. Various types of chiral compounds were efficiently separated under normal and reversed phase mode. Moreover, chloroform and tetrahydrofuran as mobile phase additives could obviously improve the resolution during the chiral separation processes. CPMs still have good chiral separation property when eluted with solvent systems with a high content of tetrahydrofuran and chloroform, which proved the high solvent resistance of this new material.

## 1. Introduction

Nowadays, the separation of enantiomers plays an increasingly important role in the fields of biology and pharmacology [1,2,3]. High-performance liquid chromatography (HPLC) using chiral stationary phases (CSPs) is considered one of the most effective methods to separate enantiomers because of the high separation efficiency and the general applicability [4,5]. Thus, new types of CSPs have been developed for enantioseparation by HPLC. Chiral metal-organic frameworks have been prepared as a new chiral packing material [6,7,8,9]. Barium-doped cyclofructan-based CSPs have been synthesized to separate chiral phosphoric and sulfonic acids [10]. Besides, a series of clicked type CSPs were prepared by thiol-ene reactions [11,12,13,14]. Thus far, the exploitation of new types of CSPs is still a research hotspot in the field of enantioseparation.

Cellulose is one of the most commonly used chiral materials to realize chiral resolution of enantiomers due to its helical structure [15,16,17,18]. Several types of CSPs based on cellulose which exhibited excellent separation performance have been developed for HPLC chiral separation by Okamoto’s group [19,20]. Nanocrystalline cellulose (NCC), generated by hydrolysis of microcrystalline cellulose (MCC), is a promising kind of nanomaterial. NCC not only retains the major properties of cellulose, including hydrophilicity and broad chemical modification capacity, but it also has some unique characteristics, such as a high degree of crystallinity, high surface area, nanometer dimensions and optical properties [21,22,23]. These properties extend the range of applications of NCC from sustainable materials and nanocomposites to medicine and life science. In particular, NCC suspensions can form a chiral nematic liquid-crystalline phase that thus can be utilized as a template to prepare chiral ordered materials, which have been applied as a new kind of chiral separation material [24,25]. In our previous work, a CSP was prepared by coating NCC derivatives on silica gel. Compared with MCC-based CSP, NCC-based CSP showed better peak shape and higher column efficiency, which indicated that NCC is an attractive material for chiral separation [26].

In recent years, organic-inorganic hybrid silica materials combining the advantages of organic and inorganic materials have attracted significant attention in the fields of separation, adsorption and catalysis [27,28,29]. These materials are suitable for use as HPLC packing materials mainly due to their large specific surface areas, ordered porosity, good mechanical and chemical stabilities, and tunable organic groups uniformly distributed in the framework [30,31,32,33,34]. To date, hybrid organosilica spheres with small molecules, like *trans*-(1*R*,2*R*)-diaminocyclohexane, β-cyclodextrin and binaphthyl groups in the framework/pore channels, have been synthesized and applied as CSPs in HPLC [35,36,37,38,39]. However, almost all reported hybrid CSPs were synthesized only introduce small molecules in inorganic materials. Polysaccharide derivatives, the most commonly used chiral selector for CSPs, are hard to apply in hybrid materials because of the difficulty of obtaining polysaccharide-silica hybrid materials with uniform spherical morphology, narrow pore size distribution, and high mechanical and chemical stabilities. Ikai and coworkers reported an efficient immobilization of polysaccharide derivatives onto silica gel via intermolecular polycondensation of triethoxysilyl groups, which used the 3-(triethoxysilyl)propyl group as a cross-linkable group [40]. On this basis, the group reported the synthesis of organic–inorganic hybrid materials using cellulose 3,5-dimethylphenylcarbamate bearing a small amount of 3-(triethoxysilyl)propyl residues and tetraethyl orthosilicate as CSP for HPLC [41]. This hybrid material exhibited high loading capacity, but eluents with high contents of chloroform (CHCl_3_) and tetrahydrofuran (THF) could not be used because of the swelling and partially solubility issues of these hybrid beads. These disadvantages hinder the application of polysaccharide-silica hybrid materials as packing materials for liquid chromatography.

These shortcomings could be overcome by making core-shell silica particles with a silica core and an organic-inorganic hybrid porous shell, which combine the high loading capacity of organic-inorganic hybrid shell and the high chemical stability of the silica core [42]. Most core-shell particles for HPLC packing materials are now prepared by a layer-by-layer self-assembly process [43,44]. This process was based on the electrostatic interaction between a charged core and and oppositely charged species to assemble single or multilayer coatings on the silica supports. After several layer-by-layer assemble steps, silica-core-porous-shell particles were obtained. However, there are few reports about the use of core-shell silica particles for the preparation of CSPs for chiral separations by HPLC [43,45]. In 2013, chiral core-shell silica microspheres with a *trans*-(1*R*,2*R*)-diaminocyclohexane bridged moiety in the mesoporous shell were reported [43]. Compared to porous silica packing materials, core-shell type stationary phases exhibit higher column efficiency and shorter retention times owing to the decrease of mass transfer resistance. Whereas, the preparation of polysaccharide silica hybrid shell-silica core particles has still been a challenging task up to now, it is envisaged that formation of a nanocellulose-silica hybrid shell on a silica support could combine the advantages of the core-shell materials, the high separation performance and give hybrid materials with a wide application range.

In this work, a novel nanocellulose hybrid core-shell CSP for HPLC was designed to further study the applications of nanocellulose in chiral separation. By using of layer-by-layer and sol-gel methods, a nanocellulose derivative was introduced into the hybrid porous shell by the copolymerization reaction of organosilica precursors, thus, nanocellulose derivative hybrid core-shell microspheres were successfully prepared. The prepared materials were characterized by scanning electron microscopy (SEM), transmission electron microscopy (TEM) and so on. Then the enantioseparation properties of this CSP were evaluated by separating various chiral compounds under normal and reversed phase mode. In addition, the effects of different mobile phases on the chiral separations were investigated in detail.

## 2. Results and Discussion

### 2.1. Morphology of the Core-Shell Particles

**Scheme 1:**
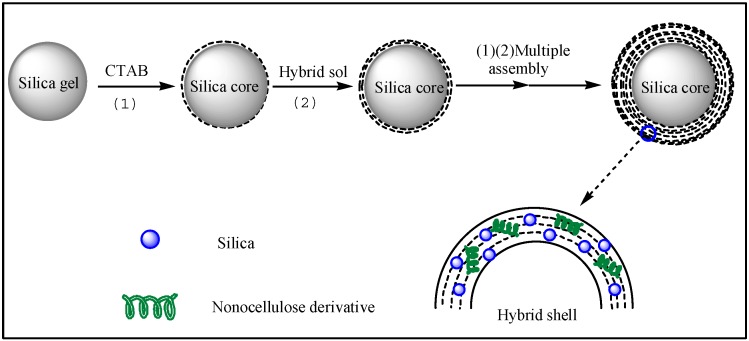
The preparation route of the core-shell particles with NCC derivatives in the hybrid shell. (1) and (2) are the assembling process of CTAB and hybrid sol, respectively.

The core-shell CPMs were prepared by a layer-by-layer self-assembly method. A surfactant monolayer was formed by electrostatic interactions on the negatively charged surface of silica particles, and the NCC derivative hybrid mesoporous layer was formed by the condensation of tetraethoxysilane (TEOS) and a NCC derivative bearing triethoxysilyl groups. The hydrolyzed silane precursor species were assembled on the surface of the silica core through S^+^I^−^ (S: surfactant; I: hydrolyzed silane precursor species) interactions under acid catalysis conditions. In this work, the effect of the content of NCC derivative on the obtained CPMs was investigated. Two CPMs with different contents of nanocellulose derivative (0.05 and 0.15 g) in the hybrid shell were prepared. These two NCC hybrid core-shell packing materials were named CPM1 and CPM2, respectively. The preparation route of the NCC hybrid core-shell particles is shown in Scheme 1.

**Figure 1:**
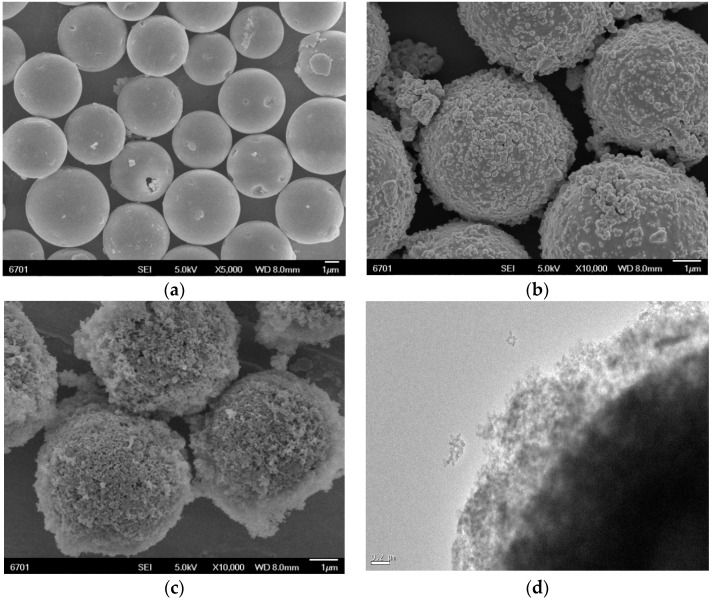
SEM images of the silica particles: (**a**) original silica particles; (**b**) CPM1; (**c**) CPM2 and (**d**) TEM images of CPM2.

The morphologies of the two core-shell CPMs with different NCC derivative content were investigated by SEM. The SEM images are shown in Figure 1. Obviously, the surface of the silica particles became rougher after the layer-by-layer self-assembly process. With the increase of the NCC derivative content in the silica-nanocellulose sol assembly process, the surface roughness of the particles and the thickness of hybrid shell increased as well. Meanwhile, when the amount of NCC derivative in each step of the hybrid sol self-assembly process was 0.15 g (Figure 1c), the shell surface was more uniform than in CPM1. This means that under the same preparation conditions, higher amounts of NCC derivative benefit the formation of hybrid shells with uniform morphology. The possible reason is that higher content of NCC derivative enhances the steric hindrance between hydrolyzed silane precursors, the self-condensation speed was slowed down and a uniform shell surface was formed. In addition, as can be seen in the TEM micrographs of CPM2 core-shell particles (Figure 1d), the shell thickness of CPM2 was about 300–400 nm.

### 2.2. Pore Structure and Compositional Characterizations of the Core-Shell Particles

The results of nitrogen adsorption/desorption measurements are listed in Table S1. As the amount of NCC derivative increased from 0.05 to 0.15 g, specific surface area of the mesoporous particles was decreased from 330 to 327 m^2^/g, and the pore volume was reduced from 0.69 to 0.58 cm^3^/g. This indicated that the hybrid shell not only coated the silica gel surface, but also the pore walls of the original silica core.

Moreover, typical N_2_ adsorption/desorption isotherms and the plot of the corresponding BJH pore size distributions of NCC hybrid core-shell particles are shown in Figure S1. The isotherms for CPM2 (Figure S1a) possessed a type IV isotherm with a hysteresis loop, which demonstrated that this material maintained a periodic mesoporous structure and a relatively narrow pore size distribution (Figure S1b).

**Figure 2:**
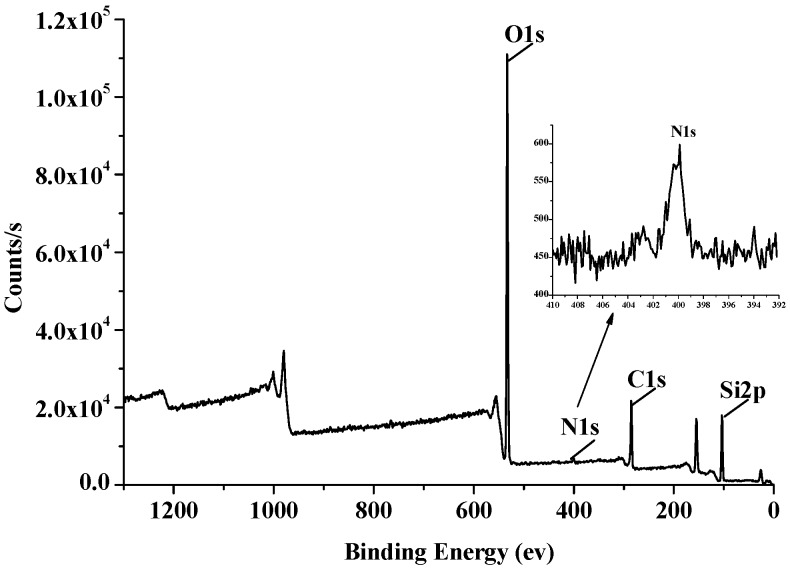
The XPS spectrum of CPM2.

In order to prove the presence of the NCC functional groups in the NCC hybrid core-shell materials, CPMs were characterized by XPS and elemental analysis measurements. Significantly, distinct O, N, C element signals are observed in the XPS spectra of CPM2 (Figure 2), which indicate that NCC derivative has been successfully introduced in the hybrid shell framework around the silica core.

Meanwhile, the elemental analysis results of the two CPMs are listed in Table 1. As the amounts of NCC derivative increased, the organic content in the hybrid core-shell material was increased. Moreover, the organic content of the core-shell CPMs were evaluated by thermogravimetric analysis and the results are shown in Figure S2. The weight loss observed at 50 °C–150 °C is due to the evaporation of water. Over 200 °C, the organic groups of CPMs start to decompose. When the amount of NCC derivative in the reaction gel increased from 0.05 to 0.15 g, the organic content of the core-shell CPM1 and CPM2 were 6.2% and 9.4%, respectively. This further indicated that a higher amount of NCC derivative was beneficial for increasing the organic content of the NCC hybrid core-shell CPMs.

### 2.3. Chiral Separation Performance

To investigate the chromatographic performance of HPLC columns packed with the CPMs, their chiral separation abilities were evaluated under normal phase and reversed phase mode with several racemates, including alcohol, ether, carboxylic acid compounds, and so on. The chromatography tests results revealed that CPM2 exhibited better recognition performance for the separation of enantiomers compared with CPM1. All of these test enantiomers were separated on CPM2 because of the high load loading of nanocellulose derivative on the hybrid shell, while only one enantiomer was partial separated on CPM1 (Figure S3). Accordingly, CPM2 was chosen to evaluate in more detail the chromatographic performance of the nanocellulose hybrid core-shell material.

#### 2.3.1. Racemic Resolutions under Normal Phase Mode

Firstly, the chromatographic performance of CPM2 was evaluated under normal phase mode. The retention factors (k), selectivity factors (α) and resolution factors (Rs) of the tested racemates are presented in Table 2. 

**Figure 3:**
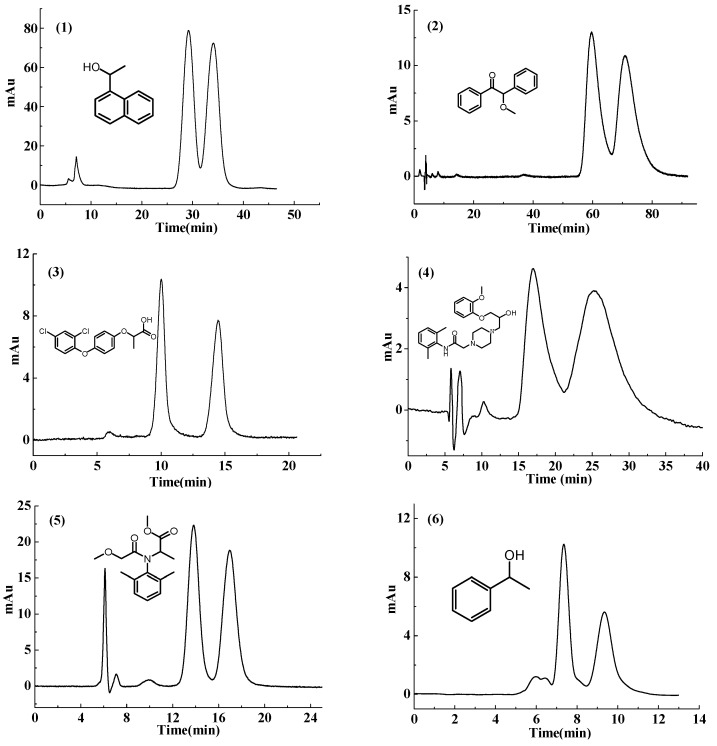
Chiral separation chromatograms of racemates on CPM2 column under normal phase mode. (**1**) 1-(1-naphthyl) ethanol; (**2**) benzoin methyl ether; (**3**) diclofop; (**4**) ranolazine; (**5**) metalaxyl; (**6**) 1-phenylethanol. Chromatographic conditions: mobile phase; (**1**–**3**) hexane/IPA (99.5/0.5, *v*/*v*); (**4**) hexane/IPA (97/3, *v*/*v*); (**5**) hexane/ethanol (97/3, *v*/*v*); (**6**) hexane/IPA/chloroform (70/15/15). Flow rate, 0.8 mL/min. Detection wavelength, 254 nm. Temperature, 25 °C.

All chromatograms for the resolution of the six racemates were depicted in Figure 3. All racemates were well enatioseparated on the CPM2 column. Among them, racemates of diclofop, metalaxyl, and 1-phenylethanol were completely separated with the Rs values of 3.17, 1.88 and 1.68. The results indirectly demonstrated that the CPM2 column has favorable chiral separation properties under normal phase mode. In addition, these racemates were separated on a commercial Chiralpak IB column under normal phase mode. The related separation parameters are also shown in Table 2. The α and Rs values of compound **6** on CPM2 column were higher than on the commercial column. The α values of compounds **1**, **2** and **3** on CPM2 were close to those of the commercial column. Meanwhile, most Rs values of the test racemates on the commercial column were higher than on the CPM2 column.

The effect of alcohol content on enantiomer separation was investigated by using different contents of isopropanol (IPA) in the separation of 1-(1-naphthyl)ethanol on a column packed with CPM2. As shown in Table S2, the k and Rs of 1-(1-naphthyl) ethanol decreased when the concentration of IPA in the mobile phase was increased. The probable reason is that, as the content of IPA increased, the eluent intensity of the mobile phase was enhanced, then the hydrogen bonding interactions between 1-(1-naphthyl)ethanol and chiral recognition sites on the CSP were weakened. Besides, the influence of hydrogen bonding interactions in the chiral separation on the CPM2 column was further studied by separating 1-(1-naphthyl)ethanol with different types of alcohols as mobile phase modifiers. As can be seen from Table S3, IPA was the optimum alcohol modifier for 1-(1-naphthyl)ethanol. Apparently, the various structures and diverse polarities of different alcohols lead to a variation in the interaction force between the alcohols and the chiral recognition sites on the CPM2 column. The above results illustrated that hydrogen bonding interactions are a significant factor in the chiral separation on a NCC derivative hybrid core-shell column.

High chemical stability is one of the most essential advantages of nanocellulose derivative hybrid core-shell stationary phases. High contents of mobile phase additives such as THF and CHCl_3_ could be used in the enantioseparation to improve peak shape and separation performance of analytes. In this work, THF and CHCl_3_ were used to improve the separation of ranolazine on the CPM2 column. The result revealed that THF had little effect on the separation of ranolazine, while CHCl_3_ is an effective additive for the resolution of ranolazine. Therefore, the effect of different contents of CHCl_3_ on enantiomer resolution has been investigated and the results are listed in Table S4. Obviously, ranolazine was not separated when using a mobile phase without additives, while it was separated after adding 15% CHCl_3_ as mobile phase additive. Hence, CHCl_3_ can be used as an effective mobile phase additive for enantioseparation on nanocellulose derivative hybrid core-shell columns.

#### 2.3.2. Racemic Resolutions under Reversed Phase Mode

**Figure 4:**
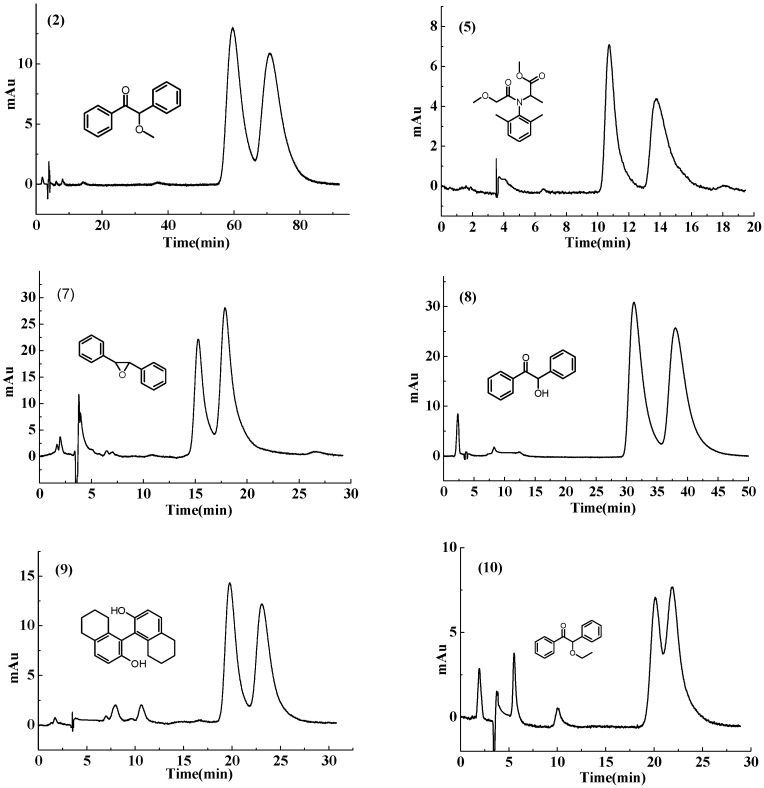
Chiral separation chromatograms of racemates on CPM2 column under reversed phase mode. (**2**) benzoin methyl ether; (**5**) metalaxyl; (**7**) *trans*-stilbene oxide; (**8**) benzoin; (**9**) octahydro binaphthol; (**10**) benzoin ethyl ether. Chromatographic conditions: mobile phase; (**2**, **8**, **10**) acetonitrile/water (15/85, *v*/*v*); (**5**, **7**) acetonitrile/water (20/80, *v*/*v*); (**9**) acetonitrile/water (30/70, *v*/*v*). Flow rate, 0.6 mL/min. Detection wavelength, 254 nm. Temperature, 25 °C.

The chromatograms for the enantioseparation of various kinds of racemates using the CPM2 column under reversed phase mode are presented in Figure 4, and the k, α and Rs of the racemates are presented in Table 3. Six racemates can be effectively separated. Among them, metalaxyl and benzoin were nearly separated by baseline, and the metalaxyl has the biggest Rs at 1.62. Moreover, the enantioseparation of these racemates were obtained on the commercial Chiralpak IB column under reversed phase mode, and the results were summarized in Table 3.

The α values of these racemates on the CPM2 column were all higher than on the commercial column. The Rs values of compounds **7** and **9** on CPM2 column were 1.13 and 1.42, respectively, which were higher than that on commercial column. The Rs values of compounds **2**, **8** and **10** on CPM2 were close to those on the commercial column, and compound **5** was better separated on the commercial column. The results indicated that the CPM2 column have certain advantages for the separation of several compounds, and CPM2 support was stable and suitable for both normal phase and reversed phase chromatographic conditions with good chiral separation performance, which further confirms that the NCC derivative has been successfully introduced in the hybrid shell of CPM2 as the chiral reorganization functional group. Therefore, NCC hybrid core-shell CPMs can be used as promising packing materials for the separation of racemates.

#### 2.3.3. Solvent Resistance of CPM2 Columns

As the result in the data presented in Section 2.3.1, mobile phase additives (such as THF, CHCl_3_ and ethyl acetate) could effectively improve the resolution of racemates during the enantioseparation. However, excessive polar solvents can damage column efficiency and separations by dissolving and swelling the polysaccharide derivatives in traditional CSPs. As reported by Okamoto, cellulose hybrid bead-type CPMs are not tolerant to mobile phases containing high concentrations of CHCl_3_ or THF. The high organic content of cellulose hybrid CPM might result in swelling or partial dissolution, which limits its application in enantioseparation tasks [35]. Hence, solvent resistance is a significant factor to evaluate the performance of CSPs. Herein, the solvent resistance of the CPM2 column was evaluated by eluting with different contents of CHCl_3_ and THF for 8 h, respectively. During this process, the column pressure was nearly constant, which indicated the column had good swelling resistance ability. After eluting with different contents of CHCl_3_ and THF, the chiral separation properties of the CPM2 column were assessed under the previous chromatographic conditions. The results, summarized in Table 4, indicated that the Rs of 1-(1-naphthyl)ethanol remained unchanged accompanied by a slight reduction in the value of α after the CHCl_3_ elution process. That is, the resolution of 1-(1-naphthyl)- ethanol can be basically considered to remain invariable after eluting with 100% CHCl_3_. Moreover, the values of α and Rs of 1-(1-naphthyl)ethanol were reduced slightly after the THF elution process. The results demonstrated that the nanocellulose derivative/silica hybrid core-shell material has good solvent resistance.

## 3. Experimental Section

### 3.1. Reagents and Materials

Microcrystalline cellulose (MCC), sodium hypochlorite solution, pyridine, triphenylchloromethane, methanol, HCl, anhydrous lithium chloride (LiCl), ammonia water, cetyltrimethylammonium bromide (CTAB), ethanol and hydrofluoric acid solution were purchased from Sinopharm Chemical Reagent Co., Ltd. (Shanghai, China). 3,5-Dimethylphenyl isocyanate, 3-(triethoxysilyl)propyl isocyanate and tetraethoxysilane (TEOS) were purchased from Gelest, Inc. (Morrisville, PA, USA). Racemic 1-(1-naphthyl)ethanol, benzoin methyl ether, diclofop, ranolazine, metalaxyl, 1-phenylethanol, *trans*-stilbene oxide, benzoin, octahydro binaphthol and benzoin ethyl ether were acquired from Sigma-Aldrich (Beijing, China). All of above reagents were of analytical grade. Beside, all solvents used in the HPLC experiment were HPLC grade and were obtained from Sinopharm Chemical Reagent Co., Ltd. Silica gel (particle size 5.0–5.5 μm, average pore diameter 10 nm, pore volumn 0.7–0.9 mL/g, surface area 300–340 m^2^/g) was purchased from Fuji Silysia Chemical Ltd. (Kasugai, Japan), while stainless steel columns (150 mm × 4.6 mm) were purchased from Hanbo Technologies (Hongkong, China). The commercial Chiralpak IB column was purchased from Daicel Chiral Technologies Co., Ltd. (Shanghai, China).

### 3.2. Instrumentations

Chromatographic separation was performed on a Waters HPLC system (Waters, Milford, MA, USA), consisting of a Waters 515 HPLC pump, a Rheodyne 7725i injector equipped with 20 μL sample loop and a Waters 2487 double λ absorbance UV detector. The separation process was carried out at 25 °C. The columns were packed by an Alltech 95551U HPLC slurry packing apparatus (Alltech, Nicholasville, KY, USA). SEM images were obtained using JSM-5600LV scanning electron microscope (JEOL, Akishima, Japan). TEM images were performed on a Tecnai G^2^-F30S-Twin at an accelerating voltage of 300 kV (FEI, Hillsboro, OR, USA). X-ray photoelectron spectroscopy (XPS) measurement was performed on a VG ESCALAB 210 instrument provided with a dual Mg/Mg anode X-ray source (Thermo, Waltham, MA, USA). Elemental analysis was performed on a Vario EL instrument (Elementar, Hanau, Germany). Thermogravimetric analysis was perfomed on a 449F3 simultaneous thermal analyzer (Netzsch, Selb, Germany).

### 3.3. Synthesis of NCC Derivatives

**Scheme 2:**
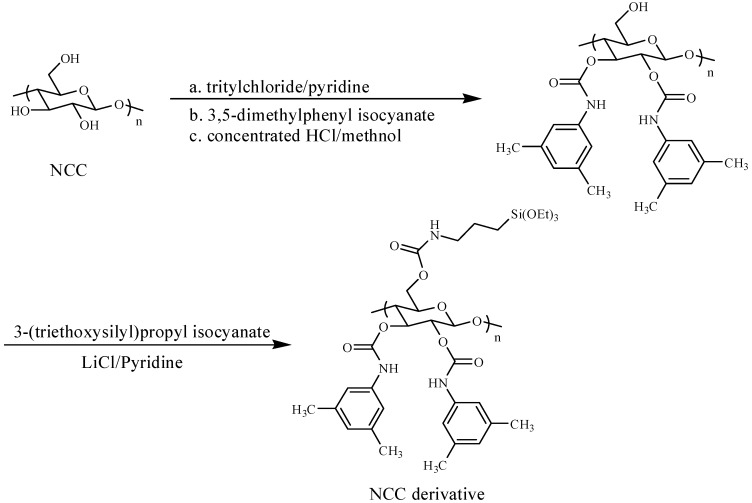
Synthetic procedure of NCC derivatives bearing a triethoxysilyl group at the 6-position and 3,5-dimethylphenyl ones at the 2- and 3-positions.

NCC was prepared according to a previously reported method [24]. NCC (1.0 g) was dispersed and stirred in the dry pyridine (60 mL) for 24 h at 90 °C, and then triphenylchloromethane (3.5 g) was added to the solution and reacted for 12 h. Then 3,5-dimethylphenyl isocyanate (4 mL) was added to the mixture which was heated for 24 h at 80 °C. The obtained product was isolated as a methanol-insoluble fraction. The white solid was filtered and washed thoroughly with dry methanol. Then the white solid was dispersed in 2% HCl solution of methanol for 24 h. After washing and drying, the obtained derivative (1.5 g) was dissolved in a mixture of anhydrous lithium chloride (1.5 g) and dry pyridine (60 mL) and stirred for 2 h. Then, 3-(triethoxysilyl)propyl isocyanate (1.2 mL) was added to reacted with the 6-hydroxyl function, and the mixture was stirred for 16 h at 80 °C. The final product was obtained by methanol precipitation. The NCC derivative was filtered and washed with methanol and then dried under vacuum, to give the final regioselectively substituted NCC derivative. The reaction is shown in Scheme 2.

### 3.4. Preparation of the Core-Shell Particles

The hybrid core-shell particles were prepared using a layer-by-layer self-assembly method for preparing the chiral packing materials (CPMs). Before the assembly process, silica particles were activated in ammonia water and concentrated hydrochloric acid, respectively. The assembly process had two major steps. First, activated silica particles (3 g) were dispersed in 0.025 M CTAB aqueous solution (100 mL) and sonicated for 30 min and then left standing for 1 h to form a CTAB monolayer film (named CTAB-silica). This was then washed carefully with deionized water and dried under vacuum for 12 h at 60 °C. Second, CTAB-silica particles were dispersed in silica-nanocellulose sol to form a nanocellulose hybrid film. The silica-nanocellulose sol was synthesized as follows: the regioselectively substituted NCC derivative was dissolved in dry pyridine (25 mL), and then a mixture of TEOS (3 mL) and ethanol (2 mL) was added (named solution A). In another flask, CTAB (0.1 g) was dissoloved in 0.037 g/mL aqueous hydrofluoric acid solution (1.0 mL), and then concentrated hydrochloric acid (0.5 mL) was added. After CTAB was completely dissolved, this mixture was added to the solution A under vigorous stirring at 15 °C. The mixture was reacted for 4 h with stirring to form stable hybrid silica sol. Then CTAB-silica was added into the hybrid silica sol for 1.5 h. After that, the silica particles were separated by centrifugation and washed thoroughly with ethanol and deionized water and dried under vacuum for 12 h at 60 °C. The whole assembly procedure was repeated eight times. Finally, the surfactant was extracted by Soxhlet extraction using 50% ethanol as extractant. To optimize the preparation condition of the nanocellulose hybrid core-shell material, two CPMs with different contents of nanocellulose derivative (0.05, 0.15 g) in the hybrid shell were prepared. These two NCC hybrid core-shell packing materials were named CPM1 and CPM2, respectively.

### 3.5. Column Packing

The prepared CPMs were slurry-packed into stainless steel columns (150 mm × 4.6 mm) under a constant packing pressure of 50 MPa with dioxane-tetrachloromethane (1:1) as slurry solvent and *n*-hexane as displacement solvent.

## 4. Conclusions

A novel chiral core-shell silica CSP with NCC derivative functional groups in the hybrid shell was successfully prepared by a layer-by-layer self-assembly method through electrostatic interactions. It was found that the amount of NCC derivative in the self-assembly procedure played a significant role in the morphology control of the resulting NCC hybrid core-shell CPMs. The chiral separation performance showed that various kinds of racemates can be separated on the NCC hybrid core-shell CSPs under both normal and reversed phase mode. Particularly, high contents of mobile phase additives could be used to improve the chiral separation performance. Moreover, CPM has high solvent resistance by eluting with a high content of THF and CHCl_3_. The results demonstrate that NCC derivative hybrid CPMs are a promising packing material for chiral separations in HPLC.

## Figures and Tables

**Table 1:** Elemental analysis data for two core-shell CPMs.

Sample	Element Content (wt %)		
	N	C	H
CPM1	0.12	2.95	0.76
CPM2	0.21	3.58	0.90

**Table 2:** Separation of racemates under normal phase mode.

No.	Racemates	CPM2 Column				Chiralpak IB Column			
		k_1_	k_2_	α	Rs	k_1_	k_2_	α	Rs
1	1-(1-Naphthyl) ethanol	3.78	4.65	1.23	1.40	1.49	2.21	1.48	3.39
2	Benzoin methyl ether	2.22	2.66	1.20	1.11	2.09	2.84	1.36	3.38
3	Diclofop	0.73	1.49	2.05	3.17	0.87	1.90	2.19	7.48
4	Ranolazine	1.79	3.17	1.76	1.28	4.07	11.12	2.73	4.99
5	Metalaxyl	0.27	0.61	2.29	1.88	3.83	10.86	2.83	8.64
6	1-Phenylethanol	1.42	1.97	1.39	1.68	0.65	0.72	1.11	0.24

Chromatographic conditions: (A) chromatographic column: CPM2. Mobile phase, (1–3) hexane/IPA (99.5/0.5, *v*/*v*), (4) hexane/IPA/ chloroform (70/15/15), (5) hexane/ethanol (97/3, *v*/*v*), (6) hexane/IPA (97/3, *v*/*v*). Flow rate, 0.8 mL/min. Detection wavelength, 254 nm. Temperature, 25 °C; (B) chromatographic column: Chiralpak IB. Mobile phase (1, 2, 3, 5, 6) hexane/IPA (90/10, *v*/*v*), (4) hexane/IPA (60/40, *v*/*v*). Flow rate, 1.0 mL/min. Detection wavelength, 254 nm. Temperature, 25 °C.

**Table 3:** Separation of racemates under reversed phase mode.

No.	Racemates	CPM2 Column				Chiralpak IB Column			
		k_1_	k_2_	α	Rs	k_1_	k_2_	α	Rs
2	Benzoin methyl ether	26.68	31.91	1.20	1.13	4.25	4.69	1.10	1.36
5	Metalaxyl	3.99	5.38	1.37	1.62	1.68	2.28	1.36	2.61
7	*trans*-Stilbene oxide	6.09	7.30	1.20	1.13	22.41	23.06	1.03	0.61
8	Benzoin	13.50	16.64	1.23	1.36	2.92	3.32	1.14	1.58
9	Octahydrobinaphthol	8.07	9.63	1.19	1.42	3.25	3.64	1.12	1.38
10	Benzoin ethyl ether	8.33	9.17	1.10	0.73	3.69	3.90	1.06	0.76

Chromatographic conditions: (A) chromatographic column: CPM2. Mobile phase, (2, 8, 10) acetonitrile/water (15/85, *v*/*v*), (5, 7) acetonitrile/water (20/80, *v*/*v*), (9) acetonitrile/water (30/70, *v*/*v*). Flow rate, 0.6 mL/min. Detection wavelength, 254 nm. Temperature, 25 °C; (B) Chromatographic column: Chiralpak IB. Mobile phase, (2, 5, 7, 8, 10) acetonitrile/water (30/70, *v*/*v*), (9) acetonitrile/water (40/60, *v*/*v*). Flow rate, 1.0 mL/min. Detection wavelength, 254 nm. Temperature, 25 °C.

**Table 4:** Separation of 1-(1-naphthyl) ethanol on CPM2 column after eluting with different contents of mobile phase additives.

CHCl_3_ or THF /hexane(*v*/*v*)	Elution with CHCl_3_		Elution with THF	
	α	Rs	α	Rs
0%	1.22	1.39	1.22	1.39
25%	1.18	1.37	1.18	1.16
50%	1.17	1.37	1.17	1.20
100%	1.18	1.39	1.16	0.99

Chromatographic conditions: mobile phase, hexane/IPA (99.5/0.5, *v*/*v*). Flow rate, 0.8 mL/min. Detection wavelength, 254 nm. Temperature, 25 °C.

## Supplementary Materials

Supplementary materials can be accessed at: http://www.mdpi.com/1420-3049/21/5/561/s1.

## Abbreviations

The following abbreviations are used in this manuscript:

CPMs	chiral packing materials
CSPs	chiral stationary phases
HPLC	high performance liquid chromatography
MCC	microcrystalline cellulose
NCC	nanocrystalline cellulose
CTAB	cetyl trimethyl ammonium bromide
