# Supplementary material for: Nanocellulose Derivative/Silica Hybrid Core-Shell Chiral Stationary Phase: Preparation and Enantioseparation Performance
Source: Molecules. 2016 May 4;21(5):561. doi: 10.3390/molecules21050561 (PMC6273020; doi:10.3390/molecules21050561)
Supplement: Supplementary file 1 [file molecules-21-00561-s001.pdf]

Supplementary Materials: Nanocellulose Derivative/Silica Hybrid Core-Shell Chiral Stationary Phase: Preparation and Enantioseparation Performance

Xiaoli Zhang, Litao Wang, Shuqing Dong, Xia Zhang, Qi Wu, Liang Zhao and Yanping Shi

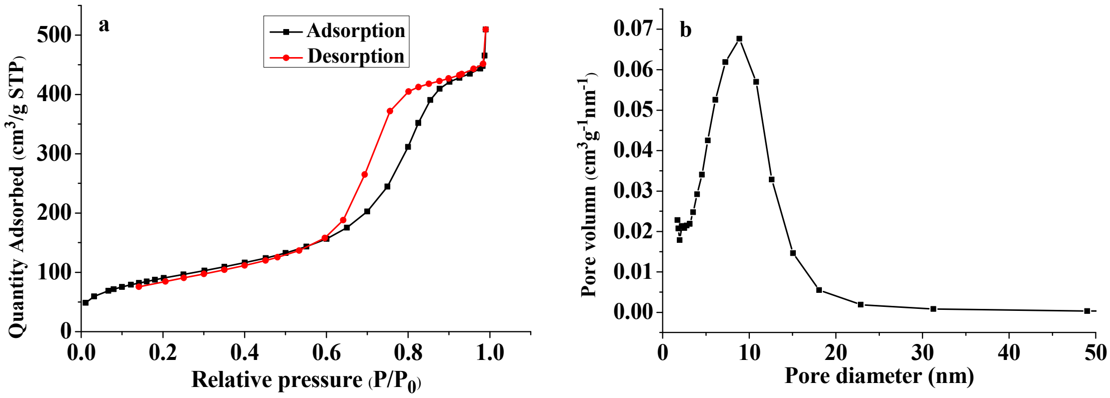

Figure S1. (**a**) N2 adsorption-desorption isotherms of CPM2; (**b**) pore size distribution of CPM2.

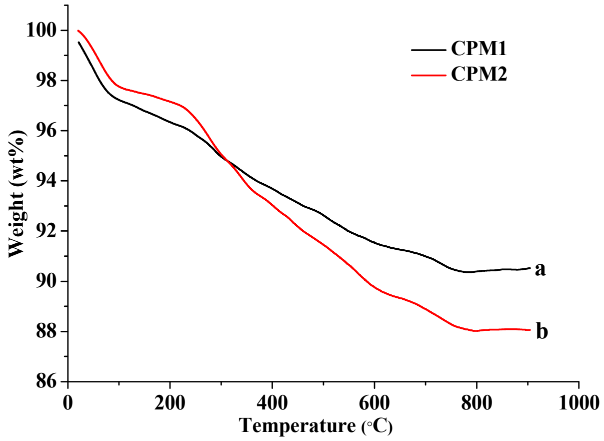

Figure S2. Thermogravimetric weight loss curves for (**a**) CPM1; (**b**) CPM2.

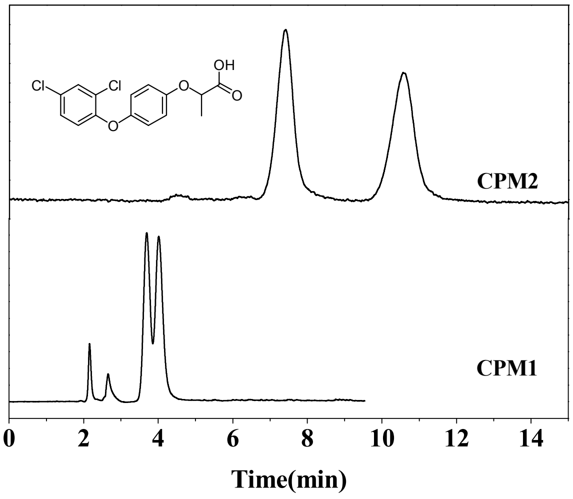

Figure S3. Chiral separation chromatograms of diclofop on columns packed with CPM1 and CPM2.

Table S1. Comparison of specific surface area, pore volume and average pore diameter of core-shell CPMs and original silica core.

	**Surface Area (m^2/g)**	**Pore Volume(cm^3/g)**	**Pore Size (nm)**
Silica core	340	0.89	10.1
CPM1	330	0.69	8.3
CPM2	327	0.58	7.1

Table S2. Effect of IPA concentration on resolution of 1-(1-naphthyl) ethanol.

**IPA (v%)**	**k_1**	**k_2**	**α**	**Rs**
0.5	3.79	4.65	1.23	1.40
1	2.48	3.05	1.23	1.25
3	1.13	1.39	1.23	1.04
10	0.35	0.41	1.17	0.53

Table S3. Effect of alcohol types on resolution of 1-(1-naphthyl) ethanol.

**Alcohol (1%, v%)**	**k_1**	**k_2**	**α**	**Rs**
n-Propanol	2.44	2.72	1.12	0.84
Butanol	2.74	3.07	1.12	0.90
Ethanol	1.75	2.14	1.22	1.20
IPA	2.48	3.05	1.23	1.25

Table S4. Effect of $CHCl_3$ on resolution of ranolazine.

**$CHCl_3$ (v%)**	**k_1**	**k_2**	**α**	**Rs**
0	8.35	8.35	1.00	--
10	2.39	3.53	1.47	1.04
15	1.79	3.17	1.76	1.28
